# Efficacy of a probiotic fermented herb in the prevention and treatment of fish nocardiosis

**DOI:** 10.3389/fvets.2025.1728878

**Published:** 2026-01-14

**Authors:** Weifu Li, Yanqi Wu, Qiuxia Su, Xu Kang, Yuhao Li, Deyu Ning, Aixuan Xu, Xinxin You, Ting Huang, Jianlin Chen, Yishan Lu

**Affiliations:** 1 Guangdong Provincial Key Laboratory of Aquatic Animal Disease Control and Healthy Culture, Fisheries College of Guangdong Ocean University, Shenzhen Institute of Guangdong Ocean University, Zhanjiang, Guangdong, China; 2 Shenzhen BGI-Marine Technology Co., Ltd., Shenzhen, Guangdong, China; 3 Guangxi Key Laboratory of Aquatic Genetic Breeding and Healthy Aquaculture, Guangxi Academy of Fishery Science, Nanning, Guangxi, China

**Keywords:** probiotic fermented herb, *Bacillus amyloliquefaciens*, fish nocardiosis, *Micropterus salmoides*, PFH

## Abstract

*Nocardia seriolae* poses a severe threat to the sustainable development in the aquaculture of a virous freshwater as well as marine fish species. In the present study, the herb No.100 (it has not been made public due to patent protection) and *B. amyloliquefaciens* MS-2 with obvious inhibitory effects against *N. seriolae* strain NS-23 were screened from 190 herbal extracts and 5 probiotic strains for developing probiotic fermented herb (PFH) against nocardiosis in largemouth bass (*Micropterus salmoides*). An ideal fermentation parameter was established by single-factor optimization as: 4% (w/v) herbal substrate with 4% (v/v) probiotic inoculum at pH 7.5 for 18 h for achieving maximum viable cell count. A 49-day feeding trial with supplements was conducted that the fish with PFH administration were significantly enhanced weight gain rates at 310.51 ± 37.44%, with increasing serum enzyme activities and nitric oxide (NO) levels, up-regulated tissue-specific expression of immune-related genes (*TNF-α*, *IL-8*, *IL-10*, *IgM*, *IFN-γ*, *IL-1β*) as well as mounting up gut microbial diversity (especially *Firmicutes* and *Bacteroidetes* species) than that of PBS control, herb or probiotic administration. Furthermore, the challenge study with *N. seriolae* strain NS-23 was conferred a 77% relative percentage survival (RPS) in the fish with PFH-supplemented diet, and the histopathological analysis confirmed that PFH effectively mitigated multi-organ necrosis and oxidative stress, leading to a complete regression of granulomas in the fish upon artificial challenge. Taken together, this PFH could be served as a safe and effective administrator against fish nocardiosis, offering a sustainable alternative to antibiotics in aquaculture.

## 1 Introduction

Aquaculture has become an indispensable component of global food production, contributing over 50% of the world’s fish supply for human consumption (1). However, the intensification of aquaculture practices has exacerbated disease outbreaks, with bacterial pathogens like *Nocardia seriolae* posing severe threats to fish health and productivity (2, 3). The natural incidence rate of fish nocardiosis caused by *N. seriolae* can reach 15 to 30%, while it reaches up to 60% in more severe cases. Under artificial infection conditions, the mortality rate can be as high as 90 to 100% (4, 5). The most distinctive clinical manifestation of fish nocardiosis is the formation of granulomatous lesions, which are pathognomonic and primarily observed in the spleen, kidney, and liver. Additionally, this bacterium with abilities in employing immune evasion strategies and establishing drug-resistant biofilm communities significantly hinders effective disease management within aquaculture systems (6, 7). Current management in fish nocardiosis heavily relies on antibiotics, but the indiscriminate use of antibiotics has fueled antimicrobial resistance and disrupted aquatic ecosystems seriously (8, 9). Consequently, the development of eco-friendly alternatives to support sustainable aquaculture in the prevention and treatment of fish nocardiosis has become a critical priority.

Medicinal herbs, as a rich source of bioactive compounds, hold great promise for disease prevention and health promotion. For examples, prenylated flavonoids isolated from *S. flavescens* disrupt the formation of pathogen biofilms by inhibiting the MAPK/NF-kB pathway (10, 11). Esculin derivatives from *F. rhynchophylla* effectively scavenge reactive oxygen species and suppress the production of pro-inflammatory cytokines (12). Additionally, limonoids derived from *M. azedarach* enhance the integrity of the intestinal barrier by regulating PTP1B (13). Despite these merits, the direct application of herbs in aquaculture remains limitation that the effective components of these herbs cannot be released effectively and be absorbed by the fish.

Fermentation technology, particularly using probiotic *Bacillus* strains, presents a transformative strategy to enhance herbal bioactivity. *B. amyloliquefaciens,* a well-documented probiotic, not only degrades antinutrients but also synthesizes bioactive metabolites (e.g., lipopeptides, organic acids) during fermentation (14). In aquaculture, *B. amyloliquefaciens* has improved growth performance in *Oreochromis niloticus* by modulating gut microbiota (15) and enhanced disease resistance in hybrid sturgeon (*Acipenser baerii* ♀ × *A. schrenckii* ♂) through immune gene regulation (16). Furthermore, fermentation of soybean meal and traditional herbs by *B. amyloliquefaciens* has been shown to elevate polysaccharide yields and antioxidant capacity, underscoring its biotransformation potential (17). However, the synergistic effects of probiotic fermented herb (PFH) with *B. amyloliquefaciens* for combating fish nocardiosis remain unexplored and it leaves a critical gap in functional feed development. Besides, previous reported studies on herbal fermentation in aquaculture often focus narrowly on growth metrics or isolated immune parameters, neglecting holistic assessments of antioxidant pathways, gut microbial ecology, and histopathological recovery. For example, while fermented astragalus (18) (*Astragalus membranaceus*) enhances *IgM* levels in *Cyprinus carpio*, its impact on oxidative stress or microbiota diversity is rarely quantified. Similarly, although *Bacillus*-fermented garlic (19) improves antioxidant enzymes in *Selenotoca multifasciata*, its efficacy against specific pathogens like *N. seriolae* remains untested. These knowledge gaps hinder the optimization of PFH for comprehensive disease management.

The present study addresses the aforementioned limitations by integrating bioprocess engineering with multi-dimensional physiological analyses. We aimed to: (1) screen herbs for developing PFH using *B. amyloliquefaciens* through single-factor parameter analysis; (2) evaluate the PFH product’s effects on growth performance, antioxidant capacity, immune gene expression, and gut microbiota structure in largemouth bass (*Micropterus salmoides*); and (3) validate PFH’s prophylactic efficacy and safety against *N. seriolae* infection through survival assays and histopathological evaluation. By elucidating the interplay between fermentation-derived metabolites, host immunity, and microbial ecology, this work provides a blueprint for developing next-generation functional feeds to replace antibiotics, addressing the intertwined challenges of disease resistance and sustainability in aquaculture.

## 2 Materials and methods

### 2.1 Medicinal herbs, bacterial strains, fish species and ethical statement

A total of 190 Chinese herbal medicines (listed in Supplementary Table S1) were all purchased from Shanxi Junkangda Biotechnology Co., Ltd. (China). The herb No. 100, which was selected for subsequent fermentation, is derived from a defined plant species within the Simaroubaceae family. It is known to be rich in characteristic Quassinoids and also contains alkaloids and flavonoids. These constituents collectively underpin its broad-spectrum antibacterial, anti-inflammatory, and immunomodulatory pharmacological activities. *N. seriolae* strain NS-23 was isolated from diseased sea bass (*Lateolabrax japonicus*) at the Hezhou North Estuary Fisheries Research Institute (Zhuhai, China) and preserved in our laboratory, which was determined as a natural virulent strain also being sensitive to largemouth bass (*M. salmoides*). *Bacillus licheniformis* strain PP789724 (MS-1), *B. amyloliquefaciens* strain PP789725 (MS-2), and *B. velezensis* strain PP789726 (MS-3) were isolated from the intestinal tracts of largemouth bass (*M. salmoides*). *B. velezensis* strain PP789722 (CA-1) and *B. subtilis* strain PP789723 (CA-2) were obtained from the intestines of northern snakehead (*Channa argus*). All strains were cryopreserved at −80 °C in our laboratory. Healthy juvenile largemouth bass (initial body weight: 15.60 ± 0.38 g) were procured from Guangdong Yangyang Fisheries Co., Ltd. (Foshan, China) and acclimatized in aerated freshwater tanks for 7 days prior to experimentation. A commercial basal diet (Qunfeng brand, crude protein: 50%, crude lipid: 10%) for largemouth bass was supplied by Guangdong Wanghai Feed Industry Co., Ltd. (Jiangmen, China). The diet was fed twice daily at 9:00 a.m. and 4:00 p.m., with a daily feeding rate of 5% of the body weight. The experimental procedures of largemouth bass were carried out in accordance with the Ethics Committee of Guangdong Ocean University (protocol code [2019] 1 and date of approval 10 May 2019).

### 2.2 Preparation and optimization of probiotic fermented herb (PFH)

#### 2.2.1 Screening of herbs and *in vitro* antibacterial assays

Each dried herb (20 g) was mixed with distilled water (1:10, w/v) and pretreated by hydration (50 °C, 30 min) to enhance permeability. A two-stage hot reflux extraction (95 °C, 30 min per cycle) was performed using a Traditional Chinese Medicine Extractor (Wuhan, China), with solvent replenishment for the second cycle. The combined extracts were concentrated to 1 g/ml, sterilized (121 °C, 15 min), and stored at 4 °C. A 100 μl suspension of *N. seriolae* strain NS-23 (OD600 = 0.6, equivalent to 1 × 10^8^ CFU/ml) was uniformly spread onto Brain Heart Infusion (BHI) agar plates. Sterile filter paper discs (6 mm diameter) impregnated with 10 μl herbal extract were aseptically placed on the agar surface. Bacteria in plate were incubated at 28 °C for 72 h, and inhibition zone diameters were measured by means of digital calipers (±0.1 mm precision).

#### 2.2.2 Screening of probiotic strains and synergistic effects

Probiotic *Bacillus* strains (MS-1, MS-2, MS-3, CA-1, and CA-2) were cultured in BHI broth at 37 °C for 24 h under aerobic conditions (150 rpm). A 100 μl suspension of *N. seriolae* strain NS-23 was spread onto a BHI agar plate, followed by incubation at 28 °C for 24 h. Sterile forceps were then used to place filter paper disks firmly onto the agar surface. A pipette was utilized to apply 10 μl of probiotic bacterial suspension onto each disk (ensuring complete saturation). Probiotic bacterial in plate were further incubated at 28 °C for 72 h, after which the diameter of the inhibition zones was measured.

#### 2.2.3 Single-factor optimization of fermentation conditions

The fermentation strains were inoculated, respectively, into the traditional Chinese medicine liquid medium with concentrations of 1, 2, 4, 6, and 8% at a 2% inoculation amount under sterile conditions. Three replicates were conducted for each concentration. After 24 h of shaking culture at 37 °C, the viable count was performed using the plate count method on BHI agar. The strain was inoculated at 1, 2, 4, 6, and 8% (v/v) into media containing the optimal herbal concentration. Post-incubation (37 °C, 24 h), the viable count was assessed. The optimal inoculation amount was inoculated onto the medium with the best herbal concentration. After being cultured on a shaker at 37 °C for 12, 18, 20, 24, 28, 32 and 36 h respectively, the viable count was assessed. Media pH was adjusted to 5.0–8.0 (0.5-unit increments) using 1 M HCl or NaOH. Post-incubation (37 °C, 24 h), the viable count was assessed. The optimal fermentation conditions were obtained in accordance with the optimal levels.

### 2.3 Effects of PFH on largemouth bass

#### 2.3.1 Experimental design and growth performance

Four experimental groups (*n* = 4 replicates/group, 40 fish/replicate) were established in a recirculating aquaculture system at Guangdong Ocean University as follows: Group A: control group, basal diet. Group B: herb group, basal diet supplemented with 8% (w/w) unfermented herb extract. Group C: probiotic group, basal diet supplemented with 8% (w/w) *B. amyloliquefaciens* MS-2 (1 × 10^8^ CFU/g feed). Group D: PFH group, basal diet supplemented with 8% (w/w) PFH. The PFH product used in Group D was standardized to contain *B. amyloliquefaciens* MS-2 at a final concentration of 1 × 10^8^ CFU/g. All the groups of fish were fed with the aforementioned supplements for 49 days, and the corresponding parameters were tested including survival rate (SR), weight gain rate (WGR), specific growth rate (SGR), feed conversion ratio (FCR), condition factor (CF), hepatosomatic index (HSI), and viscerosomatic index (VSI) in accordance with the formulas (list in Appendix A).

#### 2.3.2 Antioxidant, and immune enzyme activities

Blood samples (*n* = 6 fish/group) were collected from the caudal vein using heparinized syringes under mild anesthesia (MS-222, 100 mg/L). Serum was separated by centrifugation (3,000 *× g*, 15 min, 4 °C) and stored at −80 °C. Activities of antioxidant enzymes (superoxide dismutase (SOD), catalase (CAT)) and non-specific immune enzymes (acid phosphatase (ACP), alkaline phosphatase (AKP), and lysozyme (LZM)), as well as the concentrations of malondialdehyde (MDA) and nitric oxide (NO), were quantified using commercial assay kits (Nanjing Jiancheng Bioengineering Institute, China).

#### 2.3.3 Immune gene expression analysis

Tissue samples (blood, liver, spleen, head kidney, and intestine) were aseptically collected from euthanized fish (MS-222 overdose), immediately homogenized in TRIzol® Reagent (TransGen Biotech, China). Total RNA was purified via chloroform-isopropanol precipitation and dissolved in RNase-free water. Reverse transcription was performed using the TransScript® First-Strand cDNA Synthesis Kit (TransGen Biotech, China) with oligo(dT)18 primers. Gene-specific primers for *TNF-α*, *IL-8*, *IL-10*, *SOD*, *IgM*, *NF-κB*, *IFN-γ*, *CAT*, and *IL-1β* genes were designed using Primer Premier 5.0 (Table 1). *β-actin* served as the reference gene. qPCR was conducted using qPCR SuperMix (TransGen Biotech, China) on a 7,500 Real-Time PCR System (Applied Biosystems, USA) under the following cycling parameters: 95 °C for 2 min; 40 cycles of 95 °C (15 s), 60 °C (10 s), and 72 °C (20 s). Relative gene expression was calculated using the 2^−ΔΔCt^ method.

**Table 1:** Primer sequences for qPCR.

Primer name	Primer sequences (5′-3′)	Purpose
qMS-*β*-actin-F	TACGCCCTGCCCCATG	qPCR
qMS-*β*-actin-R	TCACGCACGATTTCCCTTT	qPCR
qMS-*TNF*-*α*-F	CTTCGTCTACAGCCAGGCATCG	qPCR
qMS-*TNF*-*α*-R	TTTGGCACACCGACCTCACC	qPCR
qMS-*IL*-*1β*-F	CGTGACTGACAGCAAAAAGAGAGG	qPCR
qMS-*IL*-*1β*-R	GATGCCCAGAGCCACAGTTC	qPCR
qMS-*IL-8*-F	TTTGGTGGAATGGGAAACTGT	qPCR
qMS-*IL-8*-R	TGTGCCTAAAGATGTAGCGAAT	qPCR
qMS-*IL-10*-F	CGGCACAGAAATCCCAGAGC	qPCR
qMS-*IL-10*-R	CAGCAGGCTCACAAAATAAACATCT	qPCR
qMS-*SOD*-F	TTTTGAGCAGGAGGGCGATT	qPCR
qMS-*SOD*-R	CCCAAGTCTCCAACATGCCT	qPCR
qMS-*IgM*-F	CTGGACCAGTCTCCCTCTGA	qPCR
qMS-*IgM*-R	CGAGGTACTGAGTGCTGCTG	qPCR
qMS-*IFN-γ*-F	AGATCAGAGGCTTTCAAATCCC	qPCR
qMS-*IFN-γ*-R	CAACATGTGGCTAATCAGCTT	qPCR
qMS-NF-κB-F	CCACTCAGGTGTTGGAGCTT	qPCR
qMS-NF-κB-R	TCCAGAGCACGACACACTTC	qPCR
qMS-CAT-F	CTATGGCTCTCACACCTTC	qPCR
qMS-CAT-R	TCCTCTACTGGCAGATTCT	qPCR

#### 2.3.4 Gut microbiota profiling

Intestinal samples (*n* = 6/group) were aseptically excised from euthanized fish, snap-frozen in liquid nitrogen and stored at −80 °C. Microbial genomic DNA was extracted using HiPure Soil DNA Kit (Magen, Guangzhou, China). The V3–V4 region of the 16S rRNA gene was amplified with universal primers 341F and 805R. Purified amplicons were sequenced on an Illumina NovaSeq 6000 platform (2 × 250 bp) by GeneDenovo (Guangzhou, China). The raw sequences were then quality-filtered, assembled, and classified against the SILVA database for taxonomic analysis.

### 2.4 Efficacy and safety of PFH against fish nocardiosis

Fish (*n* = 30/group) were intraperitoneally injected with 100 μl of *N. seriolae* strain NS-23 suspensions (1 × 10^4^ ~ 1 × 10^8^ CFU/ml in sterile PBS). Mortality was recorded daily for 14 days, and the LD_50_ value was calculated using Probit analysis (SPSS 23.0). Following the 49-day feeding trial, fish (*n* = 60/group) were injected with 2.59 × 10^5^ CFU/ml *N. seriolae* strain NS-23 (LD_50_ dose). Survival rates and clinical symptoms were monitored for 14 days post-infection.

Survival Rates (%) = (Total number − Number of Deaths)/Total number × 100. Tissues (intestine, liver, spleen, head kidney) from surviving fish (*n* = 3/group) were separated and fixed in 10% neutral buffered formalin, embedded in paraffin, sectioned (5 μm thickness), and stained with hematoxylin and eosin (H&E) by Servicebio Co., Ltd. (Wuhan, China). Histopathological changes were examined under light microscopy.

### 2.5 Statistical analysis

Inhibition zone diameters are presented as means ± standard error of the mean (SEM) of triplicate vaccination. Growth performance, enzyme activities, and gene expression data were analyzed by one-way ANOVA followed by Duncan’s multiple range test (SPSS 23.0). Survival curves were compared using the Kaplan–Meier method with log-rank tests. Significance was set at *p* < 0.05.

## 3 Results

### 3.1 Preparation and optimization of probiotic fermented herb (PFH)

The antibacterial effects of different single Chinese herbs against *N. seriolae* strain NS-23 exhibited significant variations (Table 2). The herbs of *Cullen corylifolium*, *Houpoea officinalis* and especially No. 100 displayed obvious bactericidal effect with inhibition zone diameters at 21.81, 29.27, 38.24 mm respectively, and they were classified as extremely sensitive since inhibition zone diameter ≥20 mm. In contrast, other tested herbs showed no obvious antibacterial activity to *N. seriolae*. Based on their antibacterial efficacy and pharmacological properties, the herb No. 100 was selected as the raw material for subsequent fermentation experiments. The antibacterial activity of five *Bacillus* strains against *N. seriolae* strain NS-23 was evaluated (Table 3), and the results showed that the MS-2 (*B. amyloliquefaciens*, PP789725) isolated from the intestine of largemouth bass (*M. salmoides*), exhibited the largest inhibition zone diameter at 45.56 mm, significantly outperforming other *Bacillus* strains MS-1, MS-3, CA-1, and CA-2 (*p* < 0.05). Consequently, strain MS-2 was selected as the fermentation strain for subsequent experiments.

**Table 2:** Antibacterial effect of Chinese herb medicine on *Nocardia seriolae* (NS-23).

Herb medicine	Inhibition zone diameter (mm)
*Cullen corylifolium*	21.81
*Houpoea officinalis*	29.27
No. 100	38.24

Inhibition zone classification: ≥20 mm (extremely sensitive), 15–19 mm (highly sensitive), 10–14 mm (moderately sensitive), <10 mm (low sensitivity).

**Table 3:** Antibacterial effects of probiotics on *Nocardia seriolae* (NS-23).

Probiotics	Inhibition zone diameter (mm)
MS-1 (*Bacillus licheniformis*, PP789724)	23.72
MS-2 (*Bacillus amyloliquefaciens*, PP789725)	45.56
MS-3 (*Bacillus velezensis*, PP789726)	42.95
CA-1 (*Bacillus velezensis*, PP789722)	38.66
CA-2 (*Bacillus subtilis*, PP789723)	8.29

Inhibition zone classification: ≥20 mm (extremely sensitive), 15–19 mm (highly sensitive), 10–14 mm (moderately sensitive), <10 mm (low sensitivity).

**Figure 1:**
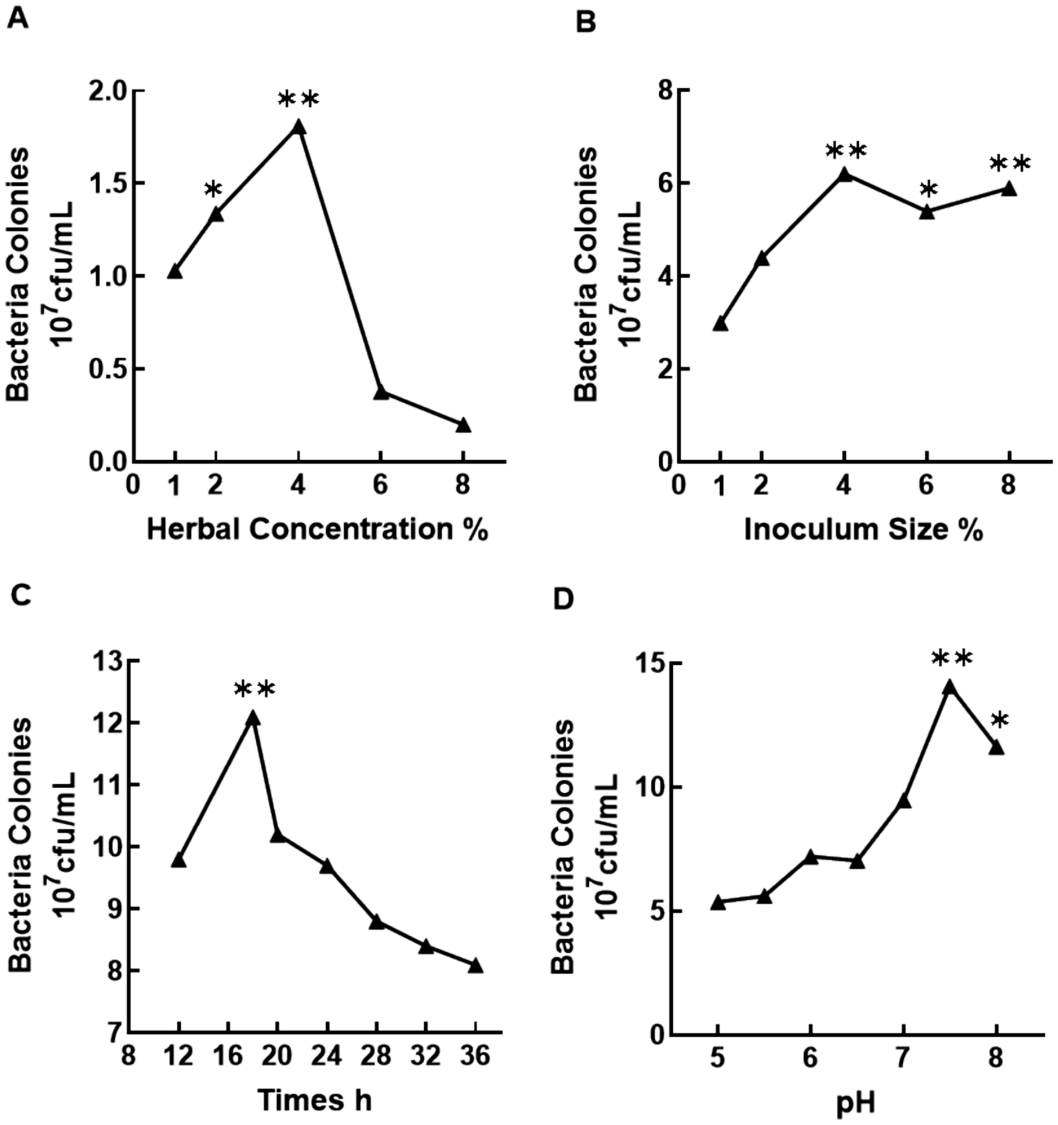
Optimization of fermentation conditions for probiotic MS-2 growth: **(A)** Effect of herb No. 100 extract concentration; **(B)** Impact of initial inoculum size; **(C)** Fermentation time analysis; **(D)** Impact of fermentation pH.

As illustrated in Figure 1, the viable cell counts of MS-2 (*B. amyloliquefaciens* strain PP789725) initially increased and then decreased with increasing herb No. 100 liquid concentration (1–8%). The optimal concentration was determined as 4%, yielding a peak viable cell count of 1.81 × 10^7^ CFU/ml, whereas 8% resulted in a significant decline (0.20 × 10^7^ CFU/ml, *p* < 0.05). Similarly, the viable cell count peaked at 6.20 × 10^8^ CFU/ml with a 4% inoculum of MS-2, declining slightly to 5.90 × 10^8^ CFU/ml at 8% (*p* < 0.05). Fermentation time and pH optimization revealed peak viable counts at 18 h (12.10 × 10^8^ CFU/ml) and pH 7.5 (14.07 × 10^8^ CFU/ml), respectively (*p* < 0.05). Thus, the optimal fermentation parameters were determined as follows: 4% herb liquid, 4% MS-2 inoculum, 18 h fermentation time, and pH 7.5.

### 3.2 Effects of PFH on largemouth bass (*M. salmoides*)

After 7 weeks of feeding, the fish in group D (PFH) exhibited significantly higher weight gain rate (WGR: 310.51 ± 37.44%) compared to the group A (277.13 ± 29.08%, *p* < 0.05). No significant differences were observed in condition factor (CF), viscerosomatic index (VSI), or hepatosomatic index (HSI) among groups (*p* > 0.05). These results indicate that PFH significantly enhances weight gain rate without adverse effects on metabolic efficiency (Table 4).

**Table 4:** Growth performance of *Micropterus salmoides.*

Parameter	Control	herb	Probiotic	PFH
SR (%)	100	100	100	100
WGR (%)	277.13 ± 29.08^b^	291.16 ± 24.82^a^	294.30 ± 27.75^a^	310.51 ± 37.44^a^
SGR (%)	2.60 ± 0.16^ab^	2.87 ± 0.05^a^	2.55 ± 0.11^b^	2.77 ± 0.18^ab^
CF (%)	1.49 ± 0.12^a^	1.49 ± 0.05^a^	1.51 ± 0.13^a^	1.51 ± 0.06^a^
VSI (%)	1.57 ± 0.38^a^	1.50 ± 0.16^a^	1.56 ± 0.26^a^	1.60 ± 0.3^a^
HSI (%)	8.23 ± 1.10^a^	7.59 ± 0.81^ab^	7.71 ± 0.55^ab^	8.12 ± 0.79^a^
FC	0.91 ± 0.01^a^	0.91 ± 0.02^a^	0.91 ± 0.01^a^	0.89 ± 0.02^a^

Values are mean ± SD (*n* = 10). Different superscript letters within a row indicate significant differences (*p* < 0.05).

**Figure 2:**
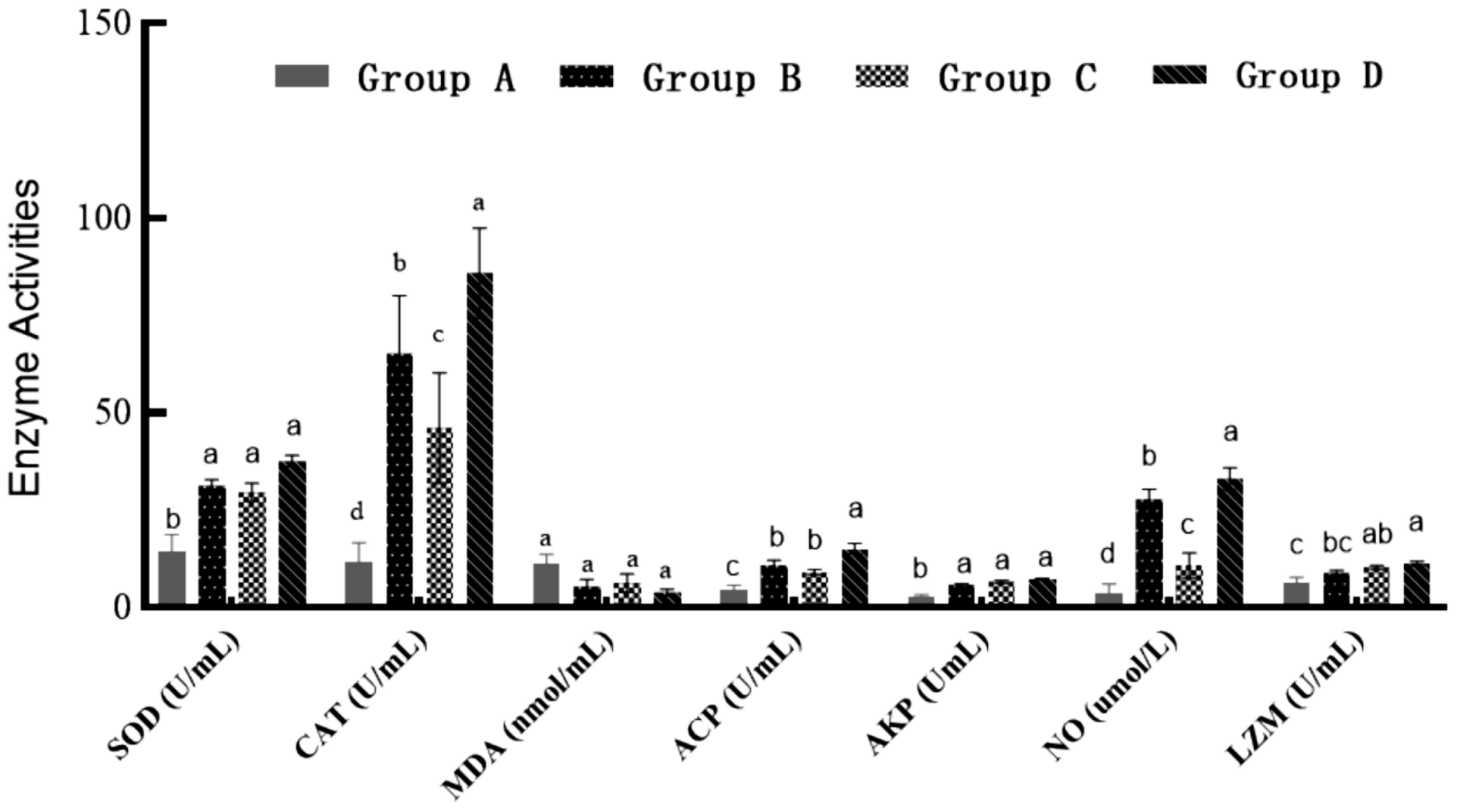
Serum antioxidant and non-specific immune parameters. SOD, Superoxide Dismutase; CAT, Catalase; MDA, Malondialdehyde; ACP, Acid Phosphatase; AKP, Alkaline Phosphatase; NO, Nitric Oxide; LZM, Lysozyme. The samples were collected 49 days after the feeding experiment. Group A: control group, basal diet. Group B: herb group, basal diet supplemented with 8% (w/w) unfermented herb extract. Group C: probiotic group, basal diet supplemented with 8% (w/w) *B. amyloliquefaciens* MS-2 (1 × 10^8^ CFU/g feed). Group D: Probiotic fermented herb (PFH) group, basal diet supplemented with 8% (w/w) PFH.

Serum superoxide dismutase (SOD) and catalase (CAT) activities in the fish of groups B, C and D were significantly higher than those in the fish of group A (*p* < 0.05, Figure 2). CAT activity followed the order: Group D > Group B > Group C (*p* < 0.05). Malondialdehyde (MDA) levels decreased in all treatment groups, but differences were not significant (*p* > 0.05). Non-specific immune enzyme activities (Figure 2) revealed that group D significantly enhanced acid phosphatase (ACP), lysozyme (LZM), and nitric oxide (NO) levels compared to group A (*p* < 0.05). Alkaline phosphatase (AKP) activity was elevated in all treatment groups (*p* < 0.05), though intergroup differences were insignificant.

**Figure 3:**
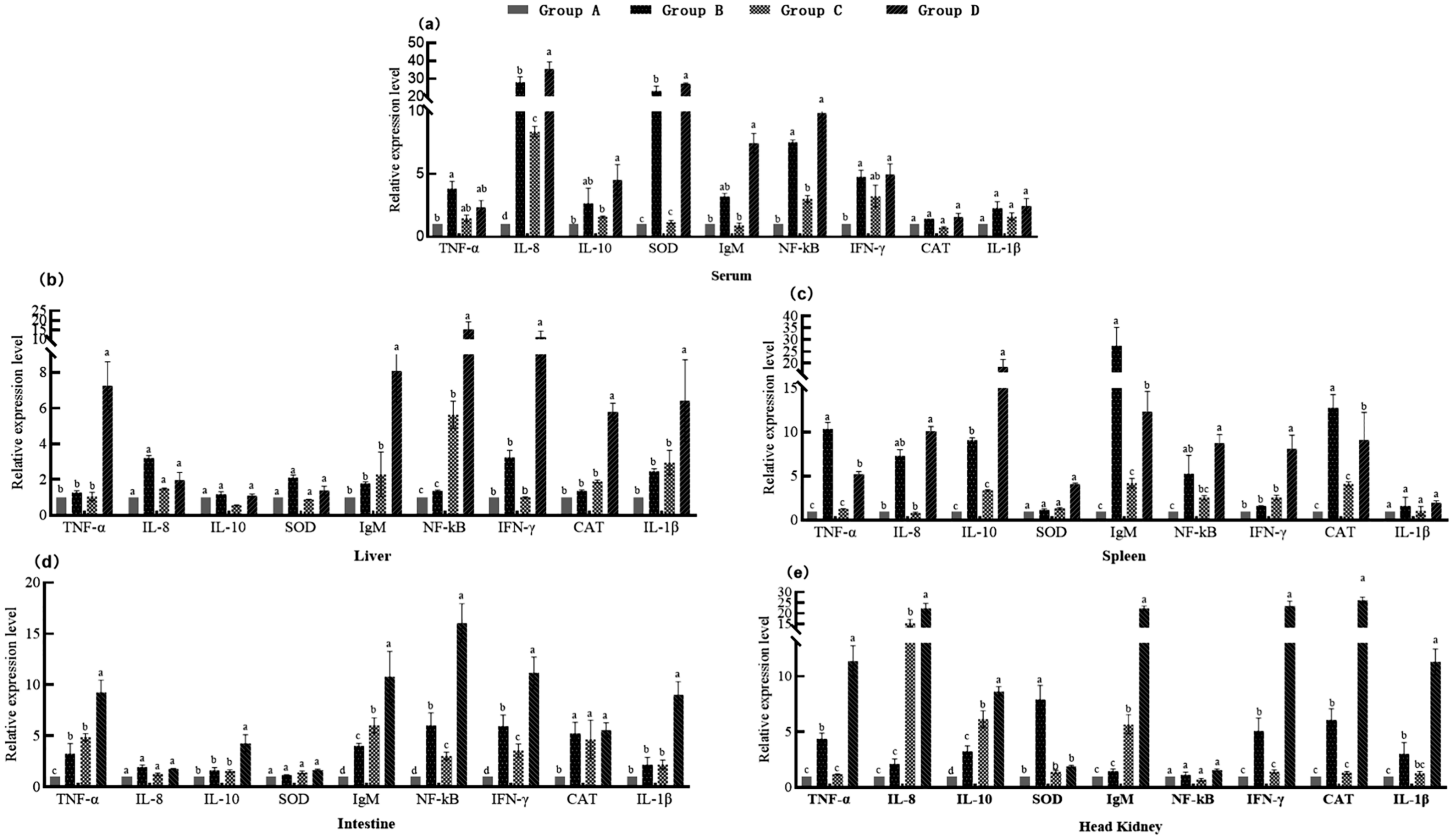
Relative expression levels of immune and antioxidant-related genes. **(a)** Blood; **(b)** Liver; **(c)** Spleen; **(d)** Intestine; **(e)** Head Kidney. Pro-inflammatory factors: *TNF-α, IL-1β, IL-8, NF-κB*; Immunoregulatory components: *IL-10, IFN-γ*; Antioxidant defense system: SOD, CAT; Humoral immunity marker: IgM; Reference gene: β-actin. The samples were collected 49 days after the feeding experiment. Group A: control group, basal diet. Group B: herb group, basal diet supplemented with 8% (w/w) unfermented herb extract. Group C: probiotic group, basal diet supplemented with 8% (w/w) B. amyloliquefaciens MS-2 (1 × 10^8^ CFU/g feed). Group D: Probiotic fermented herb (PFH) group, basal diet supplemented with 8% (w/w) PFH.

The upregulated expression of several genes related to inflammatory, antioxidant, and humoral immune genes including *TNF-α*, *IL-8*, *IL-10*, *SOD*, *IgM*, *NF-κB*, *IFN-γ*, *CAT*, and *IL-1β* genes across tissues (liver, spleen, head kidney, and intestine) were observed in the fish of group D (Figure 3). The fish of group B were showed significantly elevation in gene expressions of *TNF-α*, *IL-8*, *IL-10*, and *CAT* in blood, spleen, and head kidney (*p* < 0.05), but lower IgM and NF-κB expression in liver and spleen compared to the fish of group D. The fish of group C exhibited limitation in immune enhancement, primarily in the intestine and head kidney. In contrast, the fish of group D were demonstrated systemic immune enhancement, significantly elevating *IL-8*, *IL-10*, *SOD*, *IgM*, *NF-κB*, and *IFN-γ* expression in blood, liver, spleen, head kidney, and intestine (*p* < 0.05).

**Figure 4:**
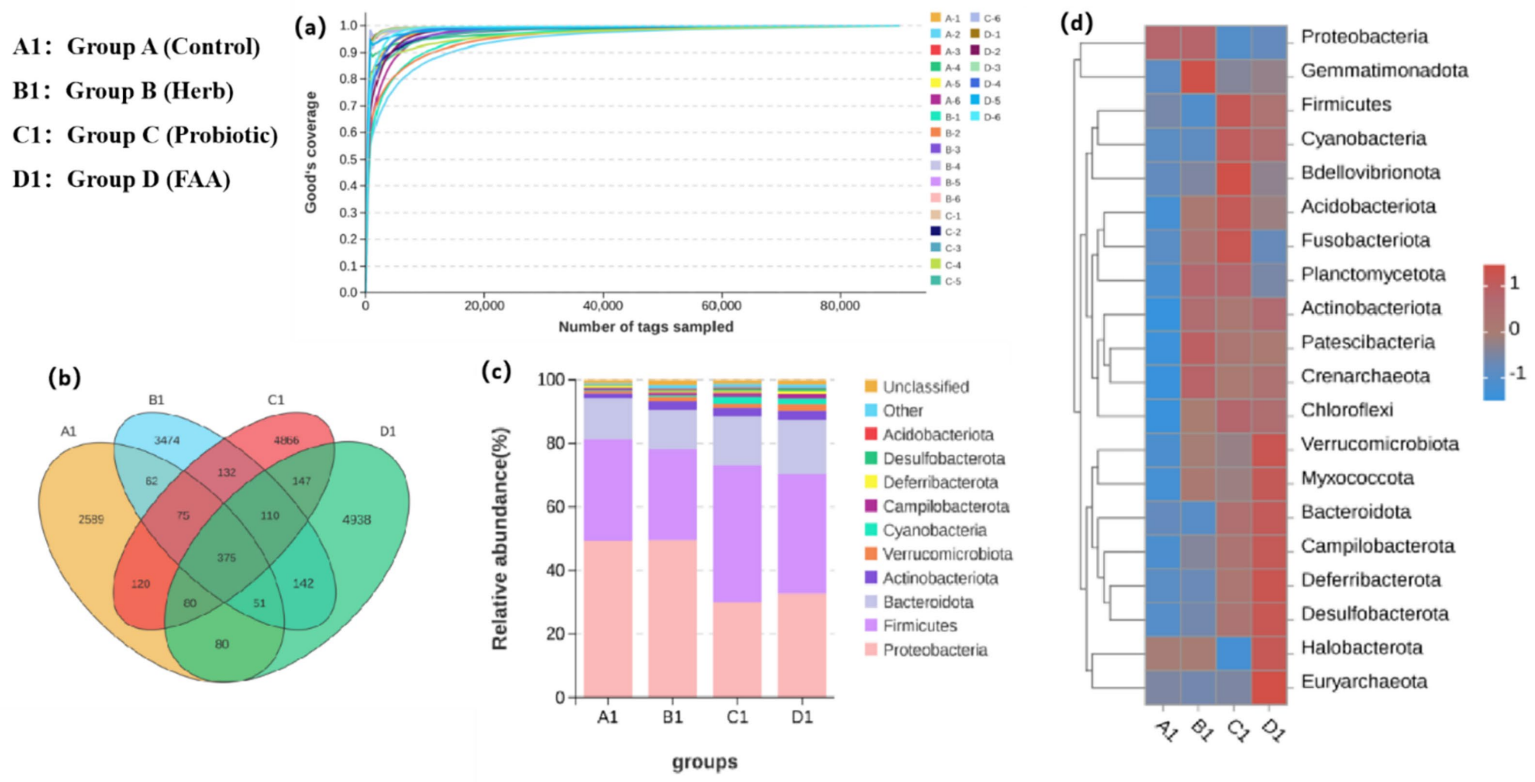
Analysis of gut microbiota profiles in *Micropterus salmoides*. **(a)** Rarefaction curves of intestinal samples; **(b)** Venn Diagram of intestinal flora species; **(c)** Stacked bar plot of intestinal microbiota abundance at the phylum level; **(d)** Heatmap analysis of species abundance.

16S rRNA sequencing revealed distinct gut microbiota profiles among different groups (Figure 4). Unique operational taxonomic units (OTUs) were identified in each group (A: 2589; B: 3474; C: 4866; D: 4938 (p < 0.05)). The relative abundance of *Proteobacteria* was highest in groups A and B, whereas *Firmicutes* represented the predominant phylum in both groups C and D. Critically, genus-level analysis demonstrated that PFH supplementation (Group D) significantly increased beneficial genera (e.g., *Lactobacillus*, *Bacillus*) while reducing potential pathogens (e.g., *Aeromonas*). This refined analysis provides a more precise understanding of the microbial restructuring induced by PFH, suggesting that the phylum-level shifts are driven by changes in specific, functionally relevant bacteria.

### 3.3 Efficacy and safety of PFH against fish nocardiosis

**Figure 5:**
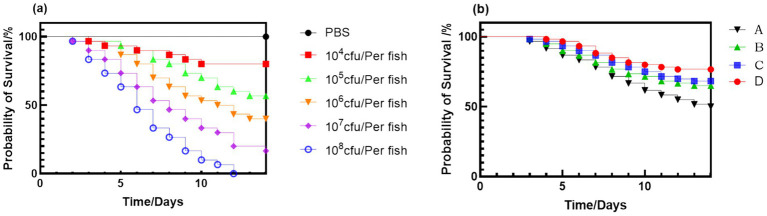
Survival response of *Micropterus salmoides* to *N. seriolae* (strain NS-23) challenge. **(a)** Dose-dependent survival curves for bacterial concentrations; **(b)** Kaplan–Meier survival curves of largemouth bass from different dietary groups after challenge with *N. seriolae* strain NS-23 at the LD₅₀ dose.

Gradient-dose challenge tests revealed dose-dependent mortality (Figure 5). The LD₅₀ was calculated as 2.59 × 10^5^ CFU/fish (Table 5). The fermented group exhibited a significantly higher immunoprotection rate of 77% (*p* < 0.05, Table 6) compared to the control group (52%), with the herb and probiotic groups achieving intermediate values of 65 and 68%. These findings collectively demonstrate the efficacy of all three interventions in enhancing host resistance against pathogens, while highlighting the superior prophylactic potential of PFH through synergistic interactions among its bioactive components.

**Table 5:** Dose-dependent mortality and LD₅₀ calculation of *N. seriolae* strain NS-23 in *Micropterus salmoides.*

Dose (CFU/fish)	Mortality (%)
1 × 10 ^8^	100
1 × 10 ^7^	83.33
1 × 10 ^6^	60
1 × 10 ^5^	43.33
1 × 10 ^4^	20
PBS	0

**Table 6:** Immune protection rate in *Micropterus salmoides.*

Group	Control	herb	Probiotic	PFH
Total (*n*)	60	60	60	60
Deaths (*n*)	29	21	19	14
Survival	31	39	41	46
Protection rate (%)	52	65	68	77

**Figure 6:**
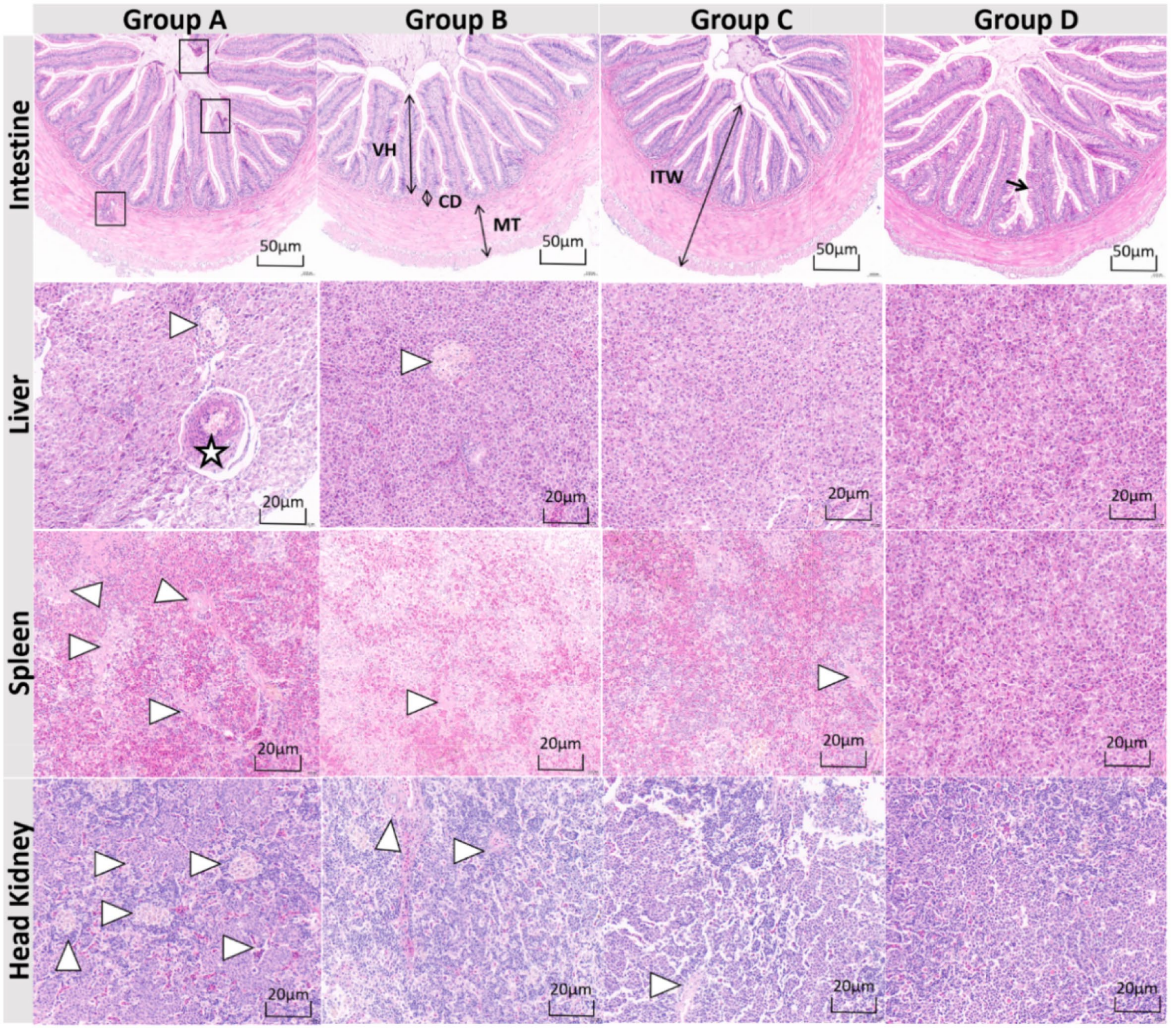
Histopathological changes. VH, villus height; CD, crypt depth; MT, muscularis thickness; ITW, intestinal wall thickness. →: goblet cells; □: inflammatory cell infiltration; ☆: granulomas; △: necrotic foci. The samples were collected 14 days after infection with *N. seriolae* (strain NS-23). Group A: control group, basal diet. Group B: herb group, basal diet supplemented with 8% (w/w) unfermented herb extract. Group C: probiotic group, basal diet supplemented with 8% (w/w) *B. amyloliquefaciens* MS-2 (1 × 10^8^ CFU/g feed). Group D: Probiotic fermented herb (PFH) group, basal diet supplemented with 8% (w/w) PFH.

Histopathological analysis (Figure 6) demonstrated significant *N. seriolae* multi-organ damage in group A, characterized by severe intestinal mucosal inflammation, hepatic granulomatous lesions, and spleno-renal necrosis, which were substantially mitigated in treatment groups. Group D exhibited superior protective efficacy, evidenced by higher villus height and intestinal wall thickness (*p* < 0.05, Table 7), and complete prevention of granuloma formation compared to group A. While groups B and C showed intermediate improvements in necrotic area reduction (35–40% vs. group A), only group D maintained near-intact histoarchitecture across all examined organs, suggesting synergistic enhancement of mucosal immunity and systemic anti-inflammatory capacity through PFH intervention strategies.

**Table 7:** Intestinal histomorphology in *Micropterus salmoides.*

Parameter	Control	Herb	Probiotic	PFH
Villus height (μm)	311.76 ± 99.16^b^	369.43 ± 54.17^b^	379.86 ± 35.76^b^	496.30 ± 78.84^a^
Intestinal wall thickness (μm)	457.84 ± 120.13^b^	523.90 ± 71.05^b^	566.29 ± 44.12^b^	637.46 ± 92.22^a^
Crypt depth (μm)	19.46 ± 2.95^b^	44.43 ± 8.68^a^	38.79 ± 6.67^a^	34.44 ± 8.83^a^
Muscularis thickness (μm)	132.65 ± 7.27^b^	138.65 ± 13.84^b^	171.78 ± 21.75^a^	98.04 ± 12.84^c^

Values are mean ± SD (*n* = 10). Different superscript letters within a row indicate significant differences (*p* < 0.05).

## 4 Discussion

### 4.1 Preparation and fermentation process optimization of probiotic fermented herb

The superior antibacterial efficacy of herb No. 100 against *N. seriolae*-demonstrating the largest inhibition zone diameter among all evaluated herbs-dictated its selection as the keystone component in the anti-nocardial formulation. This decision is pharmacologically anchored in the root’s rich repertoire of >30 quinolizidine alkaloids (e.g., matrine, oxymatrine) and >120 flavonoids (e.g., kurarinone, sophoraflavanone G) (20, 21), which concurrently disrupt bacterial membranes via lipid peroxidation, inhibit protein synthesis through 30S ribosomal subunit binding, and suppress virulence gene expression (22). *B. amyloliquefaciens* has been shown to significantly enhance intestinal health in piglets by activating the Nrf2/Keap1 signaling pathway (23). Its fermentation products exhibit antibacterial, antioxidant, and anti-inflammatory activities, effectively improving intestinal barrier function and reducing oxidative stress levels (14). In aquaculture, *B. amyloliquefaciens* has demonstrated dual benefits in hybrid sturgeon (16) and Nile tilapia (15). It not only enhances resistance to *Aeromonas hydrophila* infection by modulating the expression of immune-related genes but also significantly improves growth performance and antioxidant capacity under high-density culture conditions, while reducing stress hormone cortisol levels. As an efficient fermentative strain, *B. amyloliquefaciens* exhibits remarkable biotransformation capabilities. For instance, its fermentation of soybean meal can reconstruct the metabolite lineage to improve fish quality (17). During the fermentation of traditional Chinese medicinal materials, the contents of astragalus polysaccharide (24) and Isatis indigotica (R, S)-geichan (25) were increased by 39.59 and 7.07% respectively, confirming its significant effect in enhancing the biological activity of the products. MS-2 (*B. amyloliquefaciens* strain PP789725) used in this study possesses unique advantages, including extreme environmental adaptability (pH 2.0–12.0, bile salt tolerance of 5.0 g/L), excellent surface properties (hydrophobicity 50–93.3%, autoagglutination rate 43.6–69.2%), and abundant metabolites (26). Its inhibition zone against *N. seriolae* strain NS-23 reached at 45.56 mm (*p* < 0.05). Combined with its antibiotic sensitivity profile, MS-2 is an ideal candidate for fermentation with both biosafety and functionality, providing a valuable resource for the development of novel feed additives and functional fermented products.

In this study, the optimal conditions for the fermentation of herb No. 100 solution by MS-2 (*B. amyloliquefaciens* strain PP789725) were established through single-factor optimization. The key fermentation parameters were identified as follows: when the concentration of herb No. 100 solution reached 4%, the viable bacterial count peaked. Excessive substrate concentration led to a 9-fold reduction in viable bacterial count, likely due to inhibitory components such as phenols. An 18-h fermentation cycle effectively avoided the risk of nutrient depletion, aligning with the growth peak of MS-2 (26). A pH at 7.5 was optimal for maintaining the highest enzyme activity (27). Mechanistic analysis revealed that substrate concentration regulated bacterial population density through the synergistic effects of nutritional and inhibitory components, forming a complementary mechanism with pH sensitivity (yield increased by 18.7% in acidic environments) (28). This is similar to the observation that flavonoid content first increased and then decreased with fermentation time in the fermentation process of *Sophora chinensis* (24). The inoculum size optimization was consistent with the solid-state fermentation process of *S. japonica* (response surface method increased product yield by 34.5%), confirming the commonality of microbial fermentation population effects (29). Compared with traditional Chinese medicine fermentation processes (e.g., 48 h for astragalus polysaccharide fermentation), the fermentation time was reduced by 62.5%, significantly improving fermentation efficiency. This optimized process provides a solid technical basis for further studies on the efficacy of fermented herb No. 100 against fish nocardiosis. It is important to note that the single-factor optimization method employed here, while effective for establishing initial fermentation parameters, does not account for potential interactions between factors such as substrate concentration, inoculum size, pH, and fermentation time. Future studies utilizing response surface methodology (RSM) would be valuable to model these interactions and further refine the process for maximum metabolite yield.

### 4.2 Effects of fermented probiotic fermented herb (PFH) on largemouth bass

In this study, the weight gain rate of group D was significantly higher than that of group A (*p* < 0.05), but it did not exceed the values observed in groups B and C. Additionally, no significant differences were observed in metabolic indices such as condition factor and hepatosomatic ratio, suggesting that the growth-promoting effect of group D was attributed to intestinal microecology and immune regulation rather than enhanced metabolic efficiency. This finding is consistent with previous studies on the fermentation of licorice root, astragalus, and Siberian ginseng herb compound using *Clostridium butyricum* (no significant change in weight gain) (30) and the co-fermentation of *Pleurotus eryngii* root waste and soybean meal (slight increase in feed utilization) (31). These results indicate that the growth-promoting effect of a single fermentation substrate is limited. In contrast, systems containing polysaccharides, sulfides, and other active components, such as fermented astragalus (32), fermented garlic powder (19), and mulberry leaf-turmeric (33), have demonstrated significant growth promotion by regulating digestive enzyme activity. The mild effect of fermented herb No. 100 in this study may be due to the metabolic balance formed by the residues of quassinoid-like inhibitory components. The core active substances favored immune activation and antioxidant properties (with significant increases in SOD/CAT activities and serum IgM levels), highlighting its potential for developing disease-resistant feeds.

This study revealed that PFH exhibited unique advantages in antioxidant and immune regulation, suggesting synergistic effects among its bioactive components. The significant increase in CAT activity indicated that the fermentation process enhanced the ability to alleviate oxidative stress damage, potentially by improving the bioavailability or efficacy of active ingredients. This effect appeared to surpass the more limited impact observed with herbs or probiotics alone. In terms of non-specific immunity, the simultaneous enhancement of ACP and LZM activities and NO levels suggests a dual role in both direct bactericidal capacity and potential immunomodulation (34). This effect is closely related to the systematic regulatory characteristics of fermentation products. After fermentation of herb No. 100, partial degradation of complex components may have increased the bioavailability of active compounds, which could subsequently contribute to the observed improvements in intestinal barrier function and microbiota structure (20). This multi-target mode of action echoes similar fermentation systems: astragalus fermentation product (ALB) (32) enhances disease resistance by upregulating resistance genes (*Mx*, *IRF-3*, *TNF-α*, *IL-1β*, and *IL-10*) and bacterial diversity. *Lentinus edodes* fermentation (LEF) (35) improved intestinal amylase activity and CAT levels, decreased liver MDA levels, and inhibited inflammatory factors (*TNF-α*) to improve metabolic health, and the fermented tea residue (TF) (36) enhanced systemic antioxidant capacity through T-AOC. Although there are significant differences in the action pathways of different fermentation substrates, their commonality lies in the multi-dimensional gain of physiological functions through the reconfiguration of metabolites. While the specific pathways (e.g., MAPK/NF-κB) were not assayed here, the coordinated upregulation of immune and antioxidant genes in our study is consistent with such modulations reported in the literature. The outstanding performance of PFH on the antioxidant-immune axis in this study provides a novel candidate for developing strategies to prevent and control aquatic diseases targeting oxidative stress and immunosuppression.

In this study, PFH exhibited significant upregulation of multiple immune-related genes. This observation suggests that fermentation products may exert a synergistic effect in modulating the host immune response through multiple pathways. The immunomodulatory mechanism of fermentation products is likely closely associated with their complex bioactive components. The fermentation process may activate microbial metabolic pathways, leading to the production of novel bioactive substances capable of regulating the host immune system (37). The upregulation of pro-inflammatory genes in fermentation products does not necessarily induce excessive inflammatory responses but may represent a moderate immune activation that enhances the host’s immune capacity. For instance, pro-inflammatory cytokines such as *TNF-α* and *IL-8* play crucial roles in the early immune response by mobilizing immune cells to the site of infection (36). This differential effect may be attributed to the specific composition of the fermentation product, experimental conditions, and the immune status of the subjects studied. For example, fermented tea residue primarily exerts anti-inflammatory effects by inhibiting inflammatory signaling pathways, such as the NF-κB pathway (36). Zhou et al. reported that fermented Chinese herbal medicine significantly increased the relative mRNA expression levels of intestinal anti-inflammatory factors *TGF-β* and *IL-10*, while decreasing the expressions of pro-inflammatory factors *IL-8*, *IL-15*, and *TNF-α*, thereby playing an immune regulatory role. In contrast, the fermentation products in this study may promote the synergistic upregulation of both pro-inflammatory genes (*TNF-α*, *IL-8*) and anti-inflammatory genes (*IL-10*, *IFN-γ*) by activating multiple signaling pathways in immune cells, such as the TLR4-mediated NF-κB pathway (34). Additionally, PFH may enable more comprehensive immune regulation by enhancing the antioxidant defense system (via NRF2-mediated SOD/CAT expression) while promoting the acquired immune response (IgM secretion). The source of PFH and fermentation conditions can also influence their immunomodulatory effects. Different fermentation strains and fermentation times may lead to distinct metabolite profiles, which in turn may affect the expression of immune genes.

At the phylum level, the relative abundances of *Firmicutes* and *Bacteroidetes* were significantly increased in group D, while the proportion of *Proteobacteria* was relatively low. *Firmicutes* are closely related to the host’s energy metabolism and nutrient absorption, and their increase can improve the host’s growth performance and health status (18). *Bacteroidetes* play a vital role in carbohydrate metabolism and the production of short-chain fatty acids (SCFAs), which possess anti-inflammatory and immunomodulatory functions (32). By introducing a wider variety of microorganisms, PFH optimize the intestinal microecology, promoting the proliferation of beneficial bacteria and inhibiting the growth of harmful bacteria. This optimization of the microbiota structure was potentially associated with a significant increase in enzyme activity and immune gene expression in group D. The significant increase in weight gain rate in group D may be related to the increase in *Firmicutes* and *Bacteroidetes*, which are microorganisms that secrete a variety of digestive enzymes and promote the breakdown and absorption of nutrients. Meanwhile, the significant upregulation of immune-related genes (such as *IL-10*, *IFN-γ*, *IgM*, etc.) in group D may be closely related to the optimization of the intestinal microbiota. These results suggest that PFH significantly enhance the growth and immune function of largemouth bass by modulating the intestinal microbiota. This approach of improving fish health through microbiota regulation provides a novel direction for the development of natural and efficient aquatic functional feeds.

### 4.3 Efficacy and safety evaluation of PFH against fish nocardiosis

In the present study, PFH had significant effects on the antioxidant capacity and immunity of largemouth bass, thereby enhancing resistance to pathogens (38). This indicates that PFH was able to significantly delay the onset and progression of the disease (39).

The results showed that the protective effect of PFH on the liver, intestinal tract, spleen, and head kidney was significant. *N. seriolae* is a pathogen that can cause granulomatous inflammation and necrotic injury in multiple organs of the host. This pathological feature is closely related to the intracellular survival strategy of the pathogen and the imbalance of the host immune response (40). The widely distributed granulomas in the liver of the control group are typical markers of nocardiosis, consistent with previous study in the liver of rainbow trout (44). The pathogen activates the NLRP3 inflammasome of macrophages through the secretion of ESAT-6-like proteins, resulting in excessive release of *IL-1β*, which drives the formation of granulomas (42). The depletion of intestinal mucosal goblet cells and intestinal villi atrophy indicates that pathogens can directly destroy the mucus barrier and promote bacterial translocation, consistent with the vicious cycle mechanism of “mucus depletion–flora imbalance” in Gram-positive bacterial infection models (43). Hematopoietic tissue necrosis was present in the spleen and head kidney, indicating chronic hemolysis and oxidative stress injury (41). The changes in goblet cell number, villus height (VH), crypt depth (CD), intestinal wall thickness (ITW), and muscularis thickness (MT) in intestinal sections reflect the repair effectiveness of different intervention strategies on the mucosal barrier (40). The number of goblet cells in groups C and D were significantly higher than that in group A, consistent with reports that bacillus products promote the repair of the intestinal mucus barrier by promoting the M2 polarization of intestinal macrophages and restoring the number and function of intestinal epithelial goblet cells via the WNT-ERK1/2 axis (45). The significant increase in VH and ITW in group D indicates that short-chain fatty acids in fermentation products promote the proliferation of crypt stem cells by activating the Wnt/*β*-catenin pathway (46). Crypt depth is an important index for evaluating intestinal structural changes (47). The significant thickening of MT in group C may enhance intestinal peristaltic function by regulating intestinal glia-smooth muscle signal transmission, thereby accelerating pathogen excretion (40). Granulomas disappeared completely group D, while focal necrosis was still present in group B, highlighting the multi-target advantage of fermentation products and indicating that unfermented plant components are difficult to effectively inhibit the activation of the NLRP3 inflammasome (47). Studies have shown that ellagic acid in fermentation products alleviates oxidative stress by blocking the assembly of inflammasomes and upregulating SOD activity, which may be the key to its high efficiency (48). In summary, considering the immune protection rate, growth performance data, and histological analysis, PFH demonstrates strong safety and efficacy in the prevention and control of fish nocardiosis.

## 5 Conclusion

In the present study, the probiotic fermented herb technology not only amplified its inherent bioactivity but also introduced synergistic metabolites that enhanced host-pathogen resilience, gut-barrier integrity, and metabolic health. By addressing antibiotic resistance and ecological sustainability concerns, probiotic fermented herb represent a groundbreaking, eco-friendly alternative for aquaculture, offering dual benefits in disease prevention and growth promotion. Future research should focus on scaling production, elucidating metabolite-host interaction mechanisms, and validating probiotic fermented herb’s applicability across diverse aquatic species and farming systems. This work establishes a paradigm for leveraging bioprocessed herbal supplements to advance sustainable aquaculture practices globally.

## Data Availability

The original contributions presented in the study are included in the article/supplementary material, further inquiries can be directed to the corresponding authors.

## Appendix A

Calculation formulas for growth performance indicators.

SR (%) = (Final fish count/Initial fish count) × 100.

WGR (%) = [(Final mean weight − Initial mean weight)/Initial mean weight] × 100.

SGR (%/day) = [(ln Final mean weight − ln Initial mean weight)/Experimental duration (days)] × 100.

FCR = Total feed intake (g)/[Final total biomass (g) + Cumulative mortality biomass (g) − Initial total biomass (g)].

CF (g/cm^3^) = 100 × [Body weight (g)/Body length^3^ (cm^3^)].

HSI (%) = [Liver weight (g)/Body weight (g)] × 100.

VSI (%) = [Viscera weight (g)/Body weight (g)] × 100.

## References

[1] Food and Agriculture Organization of the United Nations. The State of World Fisheries and Aquaculture 2024. Towards Blue Transformation. Rome: FAO (2024).

[2] Chen HX Zhu YJ Dong BL Zhu ZD Cai TQ Song JP . Research progress on fish nocardiosis. Sci Fish Farming. (2021) 3:48–51. doi: 10.14184/j.cnki.issn1004-843x.2021.03.025

[3] Nguyen VV Rodkhum C Senapin S Dong HT. Retrospective diagnosis of archived marine fish experienced unexplained mortality reveals dual infections of *Nocardia seriolae* and *Streptococcus iniae*. Aquaculture International. (2019) 27:1503–12. doi: 10.1007/s10499-019-00403-4

[4] Félix A Belinda V Luis M . Phylogenetic reconstruction, histopathological characterization, and virulence determination of a novel fish pathogen *Nocardia brasiliensis*. Aquaculture. (2024) 581:740458

[5] Liu Y Chen G Xia L Lu Y. A review on the pathogenic bacterium *Nocardia seriolae*: aetiology, pathogenesis, diagnosis and vaccine development. Reviews in aquaculture. (2023) 15:14–34. doi: 10.1111/RAQ.12691

[6] Han HJ Kwak MJ Ha SM Yang SJ Kim JD Cho KH . Genomic characterization of *Nocardia seriolae* strains isolated from diseased fish. Microbiologyopen. (2019) 8:e00656. doi: 10.1002/mbo3.656 30117297 PMC6436429

[7] Xia LQ Wang M Lai JB . Establishment of a Zebrafish Model for *Nocardia seriolae* Infection and Histopathological Study. Journal of Tropical Biology. (2016) 7:409–16.

[8] Chen CF Hu M Fang P . Drug Susceptibility and Field Treatment Efficacy of Pathogens Causing Nocardiosis in Northern Snakehead (*Channa argus*). Contemporary. Aquaculture. (2014) 9:78–79,83.

[9] Jiang YY Li YW Zhou SM . Isolation and Identification of the Pathogen Causing Nocardiosis in California Bass (*Micropterus salmoides*). Journal of Sun Yat-sen University (Natural Sciences). (2012) 51:76–81.

[10] Lin Y Tu B Liao S He M. Analysis of Lavandulyl Flavonoids from *Sophora flavescens* with Antiinflammatory Activity Based on Molecular Network Technology. Medicinal Plant. (2024) 16:1–14. doi: 10.19601/J.CNKI.ISSN2152-3924.2024.02.001

[11] Kong S Liao Q Liu Y Luo Y Fu S Lin L . Prenylated flavonoids in *Sophora flavescens*: A systematic review of their phytochemistry and pharmacology. The American Journal of Chinese Medicine. (2024) 52:1087–135. doi: 10.1142/S0192415X24500447 38864547

[12] Cai T Cai B. Pharmacological activities of esculin and esculetin: A review. Medicine. (2023) 102:e35306. doi: 10.1097/MD.0000000000035306 37800835 PMC10553009

[13] Zhang SN Huang L Ma RJ . Chemical constituents from the barks of *Melia azedarach* and their PTP1B inhibitory activity. Natural Product Research. (2021) 35:4442–7. 32081038 10.1080/14786419.2020.1729146

[14] Tong Y Guo H Abbas Z Zhang J Wang J Cheng Q . Optimizing postbiotic production through solid-state fermentation with Bacillus amyloliquefaciens J and Lactiplantibacillus plantarum SN4 enhances antibacterial, antioxidant, and anti-inflammatory activities. Frontiers in Microbiology. (2023) 14:1229952. doi: 10.3389/FMICB.2023.1229952 37744928 PMC10512978

[15] Shija MV Zhimin J Chen H Amoah K Li Y Ng’onga L . Effects of dietary inclusion of Bacillus amyloliquefaciens AV5 on growth performance, antioxidant activity, innate immune, and hematological responses in Nile tilapia (Oreochromis niloticus) reared at low and high stocking densities. Fish & Shellfish Immunology. (2024) 156:110042. doi: 10.1016/J.FSI.2024.110042 39592029

[16] Su Q Peng X Zhang Z Xiong Z He B Chu P . Isolation and characterization of Bacillus subtilis and Bacillus amyloliquefaciens and validation of the potential probiotic efficacy on growth, immunity, and gut microbiota in hybrid sturgeon (Acipenser baerii ♀ × Acipenser schrenckii ♂). Fish & Shellfish Immunology. (2024) 157:110081. doi: 10.1016/J.FSI.2024.110081 39653179

[17] Wang Z Dong XW Qiao F . Bacillus amyloliquefaciens SS1-fermented soybean meal influenced the flesh texture of Nile tilapia: Involvement of microbial metabolite propionate. Aquaculture. (2025) 596:741855

[18] Chen X Liu S Teame T Luo J Liu Y Zhou Q . Effect of Bacillus velezensis T23 solid-state fermentation product on growth, gut and liver health, and gut microbiota of common carp (Cyprinus carpio). Aquaculture. (2025) 596:741733. doi: 10.1016/J.AQUACULTURE.2024.741733

[19] Basuini EFM Shaban AEM Hais EMA Soliman AA Abu Elala NM I I. Exploring the Dual Benefits of Fermented and Non-Fermented Garlic Powder on Growth, Antioxidative Capacity, Immune Responses, and Histology in Gray Mullet (Liza ramada). Fishes. (2024) 9:401. doi: 10.3390/FISHES9100401

[20] Wang ZY Wang LL Zhang J. Advances in Research on Chemical Constituents, Pharmacological Effects and Processing Methods of Sophora Flavescens. Chinese Journal of Veterinary Drug. (2019) 53:71–9.

[21] Yang RN Yao LQ Wang W . Biological functions of Sophora flavescens and its application in animal production. Animal and Feed. (2024) 23:23–6.

[22] Ma ZJ Cai JS Du ZL Li J Zhang ZC Chen F. Research progress on anti-inflammatory activity of matrine alkaloids. Modern Animal Husbandry and Veterinary Medicine. (2023) 12:63–6. doi: 10.20154/j.cnki.issn1672-9692.2023.12.014

[23] Wang Q Jin Q Wang F . *Bacillus amyloliquefaciens* SC06 alleviates LPS-induced intestinal damage by inhibiting endoplasmic reticulum stress and mitochondrial dysfunction in piglets. International journal of biological macromolecules. (2024) 282:137307 39510464 10.1016/j.ijbiomac.2024.137307

[24] Hou MR Liu Y Wang Y . Changes in active components of Astragalus during solid-state fermentation by Bacillus amyloliquefaciens. Chinese Journal of Veterinary Medicine. (2017) 53:64–8.

[25] Yin JY Liu QJ Liu XS Gao SY Zhang Y Wang LK . Effects of Bacillus amyloliquefaciens fermentation on (R,S)-goitrin content in Isatis indigotica. Modern Animal Husbandry and Veterinary Medicine. (2022) 10:7–10.

[26] Zhao HH Kang X Huang ZW . Isolation and identification of intestinal probiotics from largemouth bass (Micropterus salmoides). Journal of Guangdong Ocean University. (2023) 43:62–8.

[27] Soghomonyan T Hambardzumyan A Mkhitaryan A Khoyetsyan L Paronyan M Izmailyan M . Obtaining and Characterizing Thermostable α-Amylases Secreted by Bacillus subtilis, Originating from Bacillus amyloliquefaciens and Bacillus subtilis. Fermentation. (2024) 10:547. doi: 10.3390/FERMENTATION10110547

[28] Long M Pei X Lu Z . Effective degradation of anthraquinones in Folium Semae with Monascus fermentation for toxicity reduce and efficacy enhancement. Heliyon. (2023) 9:e18735 37560635 10.1016/j.heliyon.2023.e18735 PMC10407211

[29] Yang M Gu C Li Q Huang Z Liu H Lai Y . Optimization of Solid-state Fermentation Conditions of Sophora japonica cv. jinhua1 by Response Surface Methodology. Medicinal Plant. (2023) 14:36–41. doi: 10.19601/J.CNKI.ISSN2152-3924.2023.05.008

[30] Meng X Cai H Li H . Clostridium butyricum-fermented Chinese herbal medicine enhances the immunity by modulating the intestinal microflora of largemouth bass (Micropterus salmoides). Aquaculture. (2023) 562:738768

[31] Xu MJ Gao RW Liang P Cai HG Yang LH Lin BJ . Pleurotus eryngii root waste and soybean meal co-fermented protein improved the growth, immunity, liver and intestinal health of largemouth bass (Micropterus salmoides). Fish & Shellfish Immunology. (2024) 149:109551–1. doi: 10.1016/J.FSI.2024.109551 38599363

[32] Xue M Zhang L Meng Y . Effect of Dietary Astragalus Fermentation Products on Growth, Intestinal Microflora and Disease Resistance in Largemouth Bass Micropterus salmoides. Journal of fish diseases. (2024) 48:e14055 39628431 10.1111/jfd.14055

[33] Ilham I Sucipto S Fujaya Y. Effects of Fermented Herbal Extract as a Phytobiotic on Growth Indices, Moulting Performance, and Feed Utilization of Juvenile Tiger Shrimp (Penaeus monodon Fabr). Fishes. (2024) 9:352.

[34] Yang BZ Zhang M Wang KJ. Role of the NF-κB signaling pathway in innate immunity of fish. Biotechnology Bulletin. (2014) 1:46–52. doi: 10.13560/j.cnki.biotech.bull.1985.2014.01.030

[35] Xu J Yu Z Liu G Li S Zhou G Wang H . Effects of Dietary Lentinus edodes Fermentation Supplementation on Digestive Enzyme Activity, Antioxidant Capacity and Morphology of the Liver and Intestine in Largemouth Bass (Micropterus salmoides) Fed High Plant Protein Diets. Fishes. (2023) 8:329–43. doi: 10.3390/FISHES8060329

[36] Mao H Xinhong Z Yachao W . Use of fermented tea residues as a feed additive and effects on growth performance, body composition, intestinal enzyme activities, and inflammatory biomarkers in juvenile largemouth bass (Micropterus salmoides). Aquaculture Reports. (2023) 31:101692

[37] Xie M Zhou W Xie Y . Effects of Cetobacterium somerae fermentation product on gut and liver health of common carp (Cyprinus carpio) fed diet supplemented with ultra-micro ground mixed plant proteins. Aquaculture. (2021) 543:736943

[38] Zhou X Wang Y Yang J Li J Wu Q Bao S . Effects of dietary fermented Chinese herbal medicines on growth performance, digestive enzyme activity, liver antioxidant capacity, and intestinal inflammatory gene expression of juvenile largemouth bass (Micropterus salmoides). Aquaculture Reports. (2022) 25:101223. doi: 10.1016/J.AQREP.2022.101269

[39] Zhou T Cai P Li J Li Z Dan X Huang X . Whole genome analysis of intestinal source Bacillus and its effect on the prevention and control of hybrid snakehead (Channa maculata ♀ × Channa argus ♂) nocardiosis. Frontiers in Marine Science. (2024) 11:1367066. doi: 10.3389/FMARS.2024.1254806

[40] He SY Wei WY Liu T . Isolation, identification, and histopathological observation of the pathogen causing lethal granulomatous disease in largemouth bass (Micropterus salmoides). Journal of Fisheries of China. (2020) 44:253–65.

[41] Zhang W Zhou K Huang L . Biological characteristics and pathogenicity comparison of Nocardia seriolae isolated from Micropterus salmoides and Channa argus. Frontiers in Veterinary Science. (2024) 11:1367066. 38659458 10.3389/fvets.2024.1367066 PMC11040683

[42] Mishra BB Moura-Alves P Sonawane A Hacohen N Griffiths G Moita LF . Mycobacterium tuberculosis protein ESAT-6 is a potent activator of the NLRP3/ASC inflammasome. Cellular microbiology. (2010) 12:1046–63. doi: 10.1111/j.1462-5822.2010.01450.x 20148899

[43] Wang D Zhang B Chen M Zeng H Zhang X Zhang Y . Comparison and evaluation of DNA vaccines against Nocardia seriolae infection in largemouth bass. Aquaculture. (2025) 596:741772. doi: 10.1016/J.AQUACULTURE.2024.741772

[44] Lei XP Geng Y Zhao RX . Isolation, identification, and pathological damage of Nocardia seriolae infection in largemouth bass (Micropterus salmoides). Journal of Yunnan Agricultural University (Natural Science). (2020) 35:635–42.

[45] Liang L Liu L Zhou W Yang C Mai G Li H . Gut microbiota-derived butyrate regulates gut mucus barrier repair by activating the macrophage/WNT/ERK signaling pathway. Clinical Science. (2022) 136:291–307. doi: 10.1042/CS20210778 35194640

[46] Kou H Liu X Hu J Lin G Zhang Y Lin L. Impact of dietary zinc on the growth performance, histopathological analysis, antioxidant capability, and inflammatory response of largemouth bass Micropterus salmoides. Fish & Shellfish Immunology. (2023) 141:109025. doi: 10.1016/J.FSI.2023.109025 37625733

[47] Wang EL Wang KY Chen DF . Pathogen isolation, identification, and drug susceptibility analysis of visceral granulomatosis in cultured northern snakehead (Channa argus). Journal of Huazhong Agricultural University. (2015) 34:90–8.

[48] Gao Y Peng K Wang Y Guo Y Zeng C Hua R . Ellagic acid ameliorates cisplatin-induced acute kidney injury by regulating inflammation and SIRT6/TNF-α signaling. Food Science and Human Wellness. (2023) 12:2232–41. doi: 10.1016/J.FSHW.2023.03.043

