# Supplementary material for: Efficacy of a probiotic fermented herb in the prevention and treatment of fish nocardiosis
Source: Front Vet Sci. 2026 Jan 14;12:1728878. doi: 10.3389/fvets.2025.1728878 (PMC12853647; doi:10.3389/fvets.2025.1728878)
Supplement: Supplementary file 1 [file Data_Sheet_1.docx]

**Supplementary material**

**Table S1 190 Chinese Herbal Medicines**

| No. | Scientific Name | No. | *Scientific Name* | *No.* | *Scientific Name* |
| --- | --- | --- | --- | --- | --- |
| 1 | *Syringa oblata* | 2 | *Panax notoginseng* | *3* | *Berberis sieboldii* |
| 4 | *Zingiber officinale* | 5 | *Smilax glabra* | *6* | *Sargentodoxa cuneata* |
| 7 | *Ficus hirta* | 8 | *Allium sativum* | *9* | *Cirsium spicatum* |
| 10 | *Lilium brownii var. Viridulum* | 11 | *Cirsium arvense var. Integrifolium* | *12* | *Coix lacryma-jobi L.var.mayuen* |
| 13 | *Cornus officinalis* | 14 | *Crataegus pinnatifida* | *15* | *Cremastra appendiculata* |
| 16 | *Homalomena occulta* | 17 | *Senecio scandens* | *18* | *Cyathula officinalis* |
| 19 | *Melia azedarach* | 20 | *Portulaca oleracea* | *21* | *Lycoperdon polycephalum* |
| 22 | *Aristolochia debilis* | 23 | *Verbena officinalis* | *24* | *Saponaria calabrica* |
| 25 | *Asparagus densiflorus* | 26 | *Arecae Pericarpium* | *27* | *Euchresta japonica* |
| 28 | *Equisetum hyemale* | 29 | *Akebia quinata* | *30* | *Rubus playfairianus* |
| 31 | *Pseudocydonia sinensis* | 32 | *Schisandra chinensis* | *33* | *Rhus chinensis* |
| 34 | *Plantaginis Semen* | 35 | *Origanum vulgare* | *36* | *Actaea cimicifuga* |
| 37 | *Rosa chinensis* | 38 | *Lindera aggregata* | *39* | *Prunus mume* |
| 40 | *Pyrrosia lingua* | 41 | *Salvia chinensis* | *42* | *Haliotidis diversicolor* |
| 43 | *Dendrobium nobile* | 44 | *Microcos paniculata* | *45* | *Gentiana cruciata* |
| 46 | *Glehnia littoralis* | 47 | *Agrimonia pilosa* | *48* | *Pulsatilla chinensis* |
| 49 | *Sinapis alba* | 50 | *Ginkgo biloba* | *51* | *Dolichos lablab* |
| 52 | *Ampelopsis japonica* | 53 | *Dictamnus dasycarpus* | *54* | *Vincetoxicum atratum* |
| 55 | *Trichosanthes kirilowii* | 56 | *Isodon rubescens* | *57* | *Scrophularia ningpoensis* |
| 58 | *Lobelia chinensis* | 59 | *Luffa cylindrica* | *60* | *Geranium wilfordii* |
| 61 | *Lycium chinense* | 62 | *Panax quinquefolius* | *63* | *Myristica fragrans* |
| 64 | *Cinnamomum cassia* | 65 | *Phyllostachys glauca* | *66* | *Bambusa tuldoides* |
| 67 | *Corydalis yanhusuo* | 68 | *Daemonorops draco* | *69* | *Equisetum arvense* |
| 70 | *Juncus effusus* | 71 | *Rhodiola rosea* | *72* | *Ophiopogon japonicus* |
| 73 | *Paeonia lactiflora* | 74 | *Gleditsia sinensis* | *75* | *Biancaea sappan* |
| 76 | *Zanthoxylum bungeanum* | 77 | *Paeonia suffruticosa* | *78* | *Pleuropterus multiflorus* |
| 79 | *Eriocaulon buergerianum* | 80 | *Yulania liliiflora* | *81* | *Commiphora myrrha* |
| 82 | *Terminalia chebula* | 83 | *Cullen corylifolium* | *84* | *Ganoderma lucidum* |
| 85 | *Lonicera Japonica* | 86 | *Kadsura heteroclita* | *87* | *Celosia cristata* |
| 88 | *Canarium subulatum* | 89 | *Celosia argentea* | *90* | *Artemisia caruifolia* |
| 91 | *Baphicacanthus cusia* | 92 | *Sophora flavescens* | *93* | *Melia toosendan* |
| 94 | *Eleutherococcus senticosus* | 95 | *Curcuma aromatica* | *96* | *Reynoutria japonica* |
| 97 | *Siraitia grosvenorii* | 98 | *Ocimum basilicum* | *99* | *Sedum sarmentosum* |
| 100 | Name protected | 101 | *Eupatorium fortunei* | *102* | *Tinospora sagittata* |
| 103 | *Fagopyrum dibotrys* | 104 | *Paecilomyces cicadae* | *105* | *Boswellia carterii* |
| 106 | *Sarcandra glabra* | 107 | *Cyrtomium fortunei* | *108* | *Rubia cordifolia* |
| 109 | *Alpinia katsumadai* | 110 | *Sarcandra glabra* | *111* | *Origanum vulgare* |
| 112 | *Capsella bursa-pastoris* | 113 | *Platycladus orientalis* | *114* | *Gardenia jasminoides* |
| 115 | *Clematis chinensis* | 116 | *Houpoea officinalis* | *117* | *Amomum villosum* |
| 118 | *Allium tuberosum* | 119 | *Cyperus rotundus* | *120* | *Curcuma phaeocaulis* |
| 121 | *Paris polyphylla var. Yunnanensis* | 122 | *Trachelospermum jasminoides* | *123* | *Phryma leptostachya subsp. Asiatica* |
| 124 | *Peucedanum praeruptorum* | 125 | *Fraxinus chinensis* | *126* | *Raphanus sativus* |
| 127 | *Neoalsomitra clavigera* | 128 | *Prunus persica* | *129* | *Prunus davidiana* |
| 130 | *Eucalyptus* spp. | 131 | *Prunella vulgaris* | *132* | *Bupleurum chinense* |
| 133 | *Codonopsis pilosula* | 134 | *Commelina communis* | *135* | *Belamcanda chinensis* |
| 136 | *Vincetoxicum pycnostelma* | 137 | *Bistorta officinalis* | *138* | *Alpinia Oxyphylla* |
| 139 | *Lygodium japonicum* | 140 | *Aralia chinensis* | *141* | *Brassica juncea* |
| 142 | *Sepiella maindroni Rochebrune* | 143 | *Lithospermum erythrorhizon* | *144* | *Ziziphus jujuba var. Spinosa* |
| 145 | *Sargassum pallidum* | 146 | *Taxillus sutchuenensis* | *147* | *Scutellaria baicalensis* |
| 148 | *Astragalus membranaceus* | 149 | *Coptis chinensis* | *150* | *Polygonatum sibiricum* |
| 151 | *Cuscuta chinensis* | 152 | *Ilex rotunda* | *153* | *Polyporus spathulatus* |
| 154 | *Chrysanthemum indicum* | 155 | *Cnidium monnieri* | *156* | *Ranunculus ternatus* |
| 157 | *Phytolacca acinosa* | 158 | *Lophatherum gracile* | *159* | *Glycine max* |
| 160 | *Allium fistulosum* | 161 | *Stellaria alsine* | *162* | *Trachycarpus fortunei* |
| 163 | *Viola philippica* | 164 | *Aster tataricus* | *165* | *Inula japonica* |
| 166 | *Senna alexandrina* | 167 | *Robinia pseudoacacia* | *168* | *Tripterygium wilfordii* |
| 169 | *Taraxacum mongolicum* | 170 | *Typha angustifolia* | *171* | *Liquidambar formosana* |
| 172 | *Ardisia escallonioides* | 173 | *Citrus reticulata* | *174* | *Phragmites communis* |
| 175 | *Rhaponticum uniflorum* | 176 | *Vitex trifolia* | *177* | *Eclipta prostrata* |
| 178 | *Santalum album* | 179 | *Torreya grandis* | *180* | *Allium macrostemon* |
| 181 | *Rheum palmatum* | 183 | *Mentha canadensis* | *183* | *Potentilla discolor* |
| 184 | *Trichosanthes kieilowii* | 185 | *Rubus idaeus* | *186* | *Dianthus superbus* |
| 187 | *Abrus pulchellus subsp. Cantoniensis* | 188 | *Alisma plantago-aquatica* | *189* | *Micranthemum micranthemoides* |
| 190 | *Combretum indicum* |  |  |  |  |
